# A review of *Rheocricotopus (Psilocricotopus) chalybeatus* species group from China, with the description of three new species (Diptera, Chironomidae)

**DOI:** 10.3897/zookeys.388.6316

**Published:** 2014-03-12

**Authors:** Wenbin Liu, Xiaolong Lin, Xinhua Wang

**Affiliations:** 1 College of Life Science, Nankai University, Tianjin 300071, China

**Keywords:** Chironomidae, *Rheocricotopus*, *chalybeatus* species group, new species, key, China

## Abstract

The *Rheocricotopus (Psilocricotopus) chalybeatus* species group from China is reviewed. Three new species, *R. (P.) brochus* **sp. n.**, *R. (P.) rotundus* **sp. n.** and *R. (P.) serratus* **sp. n.** are described as adult males. *R. (P.) imperfectus* Makarchenko & Makarchenko, 2005, *R. (P.) robacki* (Beck & Beck, 1964) and *R. (P.) valgus* Chaudhuri & Sinharay, 1983 are recorded from China for the first time and annotated. The diagnosis for the species group is emended and a key to adult males of the species group in China is presented.

## Introduction

The genus *Rheocricotopus* was erected by Thienemann and Harnisch (1932). Originally, Edwards (1929) treated it as a group of the genus *Spaniotoma* Philippi, 1865. Brundin (1956) reaffirmed the valid generic status of *Rheocricotopus* to be followed by Lehmann (1969) and other workers. It can be separated from other orthoclad genera by the following combination of characters: hairy eyes; without dorsomedial extension; developed pulvilli; acrostichals beginning near antepronotum; plate-like superior volsella and pointed anal point with posterolaterally directed setae. The immature stages of *Rheocricotopus* can be collected in streams and rivers, rarely in the littoral zone of lakes (Cranston et al. 1989). So far, 70 species (Ashe and O’Connor 2012) were recorded in all zoogeographic regions in the world.

Sæther (1985) reviewed the genus *Rheocricotopus* Thienemann & Harnisch, 1932 in the world and divided the genus into two subgenera (*Rheocricotopus* *sensu stricto* and *Psilocricotopus* Sæther) including six species groups (*atripes* species group, *chalybeatus* species group, *godavarius* species group, *tuberculatus* species group, *fuscipes* species group and *effusus* species group). Wang and Sæther (2001) erected *orientalis*, a new species group. The *Rheocricotopus chalybeatus* species group can be distinguished from other species groups by the following combination of characters: gonostylus either with pronounced, preapical, triangular crista dorsalis or with apically sharp upward bend fused with apparent crista dorsalis; humeral pit moderately large, ovoid or circular, if large and somewhat rectangular gonostylus bent sharply upwards distally; superior volsella rounded, relatively small, never with projection. To date, 22 species were recorded in the *chalybeatus* species group (Sæther 1985; Caspers 1987; Chaudhuri and Sinharay 1983; Hazra and Chaudhuri 2004; Johannsen 1932; Makarchenko and Makarchenko 2005; Sasa 1990, 1991; Sasa and Suzuki 2000; Wang and Zheng 1989, 1991; Wang et al. 2004).

In China, 6 species of *chalybeatus* species group [*Rheocricotopus (Psilocricotopus) emeiensis* Wang & Zheng, 1989, *Rheocricotopus (Psilocricotopus) nigrus* Wang & Zheng, 1989, *Rheocricotopus (Psilocricotopus) bifasciatus* Wang & Zheng, 1991, *Rheocricotopus (Psilocricotopus) brachypus* Wang & Zheng, 1991, *Rheocricotopus (Psilocricotopus) chalybeatus* (Edwards, 1929) and *Rheocricotopus (Psilocricotopus) taiwanensis* Wang et al., 2004] had been recorded (Wang 2000, Wang et al. 2004).

Based on specimens from China, in this paper, three new species are described, and a key to the Chinese species of *chalybeatus* group is presented.

## Materials and methods

The morphological nomenclature follows Sæther (1980). The material examined was mounted on slides following the procedures outlined by Sæther (1969). The specimens examined in this study are deposited in the College of Life Sciences, Nankai University, China.

## Taxonomy

### Rheocricotopus (Psilocricotopus) bifasciatus

Wang & Zheng, 1991

http://species-id.net/wiki/Rheocricotopus_bifasciatus

Rheocricotopus bifasciatus Wang & Zheng, 1991: 100.
Rheocricotopus (Psilocricotopus) bifasciatus Wang, 2000: 639, Ashe and O’Connor 2012: 560.

#### Specimens examined.

**Type material:** Holotype, ♂, Chongqing City, Jinfo Mountain, 29°01'90"N, 107°16'20"E, 9.v.1986, sweeping, Wang XH. Paratype (1): 1♂, as holotype.

**Additional material:** 1♂, Sichuan Province, Yajiang County, 30°15'00"N, 101°02'00"E, 14.vii.1997, sweeping, Wang XH; 1♂, Hunan Province, Taoyuan County, 28°63'72"N, 111°13'79"E, 17.vii.2004, sweeping, Yan CC; 3♂♂, Ningxia Hui Autonomous Region, Jingyuan County, 35°66'33"N, 106°29'08"E, 7.viii.1987, sweeping, Wang XH; 1♂, Ningxia Hui Autonomous Region, Jingyuan County, 35°66'33"N, 106°29'08"E, 8.viii.1987, sweeping, Wang XH; 1♂, Gansu Province, Yuzhong County, 35°90'00"N, 104°11'00"E, 4.viii.1993, sweeping, Bu WJ.

#### Diagnosis.

This species can be separated from other members of the group by the following combination of characters: tergites I, II and IV pale brown, others dark brown; AR 0.90; wing anal lobe reduced; humeral pit large, ovoid; Costal extension 83 μm long.

#### Remarks.

The additional specimens are similar to the description of Wang and Zheng (1991). The species is recorded from Palearctic Region for the first time.

#### Distribution.

China (Chongqing Municipality, Sichuan, Hunan and Gansu Provinces, Ningxia Hui Autonomous Region).

### Rheocricotopus (Psilocricotopus) brachypus

Wang & Zheng, 1991

http://species-id.net/wiki/Rheocricotopus_brachypus

Rheocricotopus brachypus Wang & Zheng, 1991: 101.
Rheocricotopus (Psilocricotopus) brachypus Wang, 2000: 639, Ashe and O’Connor 2012: 560.

#### Specimens examined.

**Type material:** Holotype, ♂, Guangdong Province, Fengkai County, Heishiding National Nature Reserve, 23°30'02"N, 111°55'01"E, 12.iv.1985, sweeping, Wang XH.

**Additional material:** 1♂, Hubei Province, Hefeng County, 29°91'00"N, 110°03'00"E, 16.vii.1999, light trap, Ji BC; 1♂, Sichuan Province, Yajiang County, 30°15'00"N, 101°02'00"E, 14.vii.1997, sweeping, Wang XH; 1♂, Xizang Autonomous Region, Bayi County, Shergmla Mountain, 29°64'07"N, 94°36'01"E, 28–30.ix.1997, yellow trap, Solhøy T & Skartveit J.

#### Diagnosis.

This species can be separated from other members of the group by the following combination of characters: AR 0.43; anal lobe developed; anal point robust.

#### Remarks.

Wang and Zheng (1991) described this species without humeral pit which as diagnostic characteristic. However, after examining the holotype, we find a medium, relatively shallow, ovoid humeral pit existing.

#### Distribution.

Oriental China (Guangdong, Hubei and Sichuan Provinces, Xizang Autonomous Region).

### Rheocricotopus (Psilocricotopus) brochus sp. n.

http://zoobank.org/74D7F9E8-E9E8-4BE0-A21B-DDEB23C88D8B

http://species-id.net/wiki/Rheocricotopus_brochus

Figs 1–6

#### Diagnosis.

The adult male can be distinguished from known species of the species group and the genus by the following combination of characters: crista dorsalis tooth-liked; tergites I, II and IV yellow, tergite III mainly yellow with a brown circular area, other tergites brown.

#### Description.

Male imago (n = 12)

Total length 1.75–2.70, 2.18 mm. Wing length 1.25–1.60, 1.33 mm. Total length/wing length 1.35–1.78, 1.68. Wing length/length of profemur 1.76–2.31, 2.04.

**Figures 1–6.**
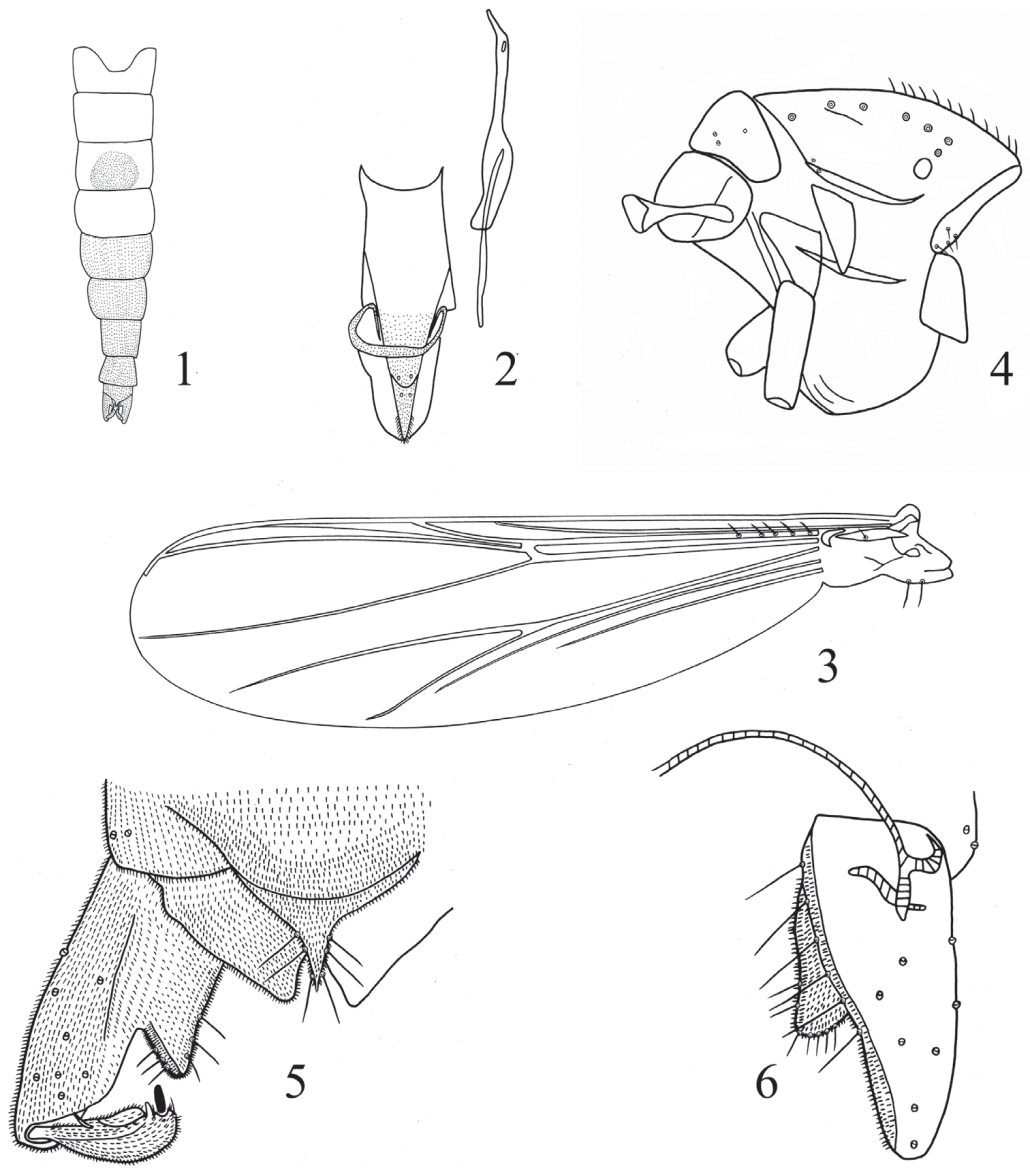
*Rheocricotopus (Psilocricotopus) brochus* sp. n., male. **1** abdomen tergites coloration **2** cibarial pump, tentorium and stipes **3** wing **4** thorax **5** hypopygium (dorsal view) **6** hypopygium (ventral view).

*Coloration*. Head and thorax brown. Tergites (Fig. 1) I, II and IV yellow, tergite III mainly yellow but having a brown circular area, other tergites brown.

*Head*. AR 0.63–0.89, 0.72. Ultimate flagellomere 245–360, 284 μm long. Temporal setae 4–7, 5, including 1–3, 2 inner verticals; 1–3, 2 outer verticals and 0–2, 1 postorbital. Clypeus with 7–10, 8 setae. Cibarial pump, tentorium and stipes as in Fig. 2. Tentorium 75–140, 123 μm long, 13–25, 19 μm wide. Stipes 100–125, 118 μm long, 8–10, 8 μm wide. Palpomere lengths (in μm): 20–25, 23; 38–55, 47; 90–130, 105; 110–138, 120; 205–250, 232. L: 5^th^/3^rd^ 1.92–2.67, 2.23. Third palpal segment with 2 sensilla clavata.

*Wing* (Fig. 3). Anal lobe reduced. VR 1.06–1.13, 1.07. Costal extension 45–65, 51 μm long. Brachiolum with 1 seta. R with 3–6, 5 setae. Remaining veins bare. Squama with 1–2, 2 setae.

*Thorax* (Fig. 4). Antepronotum with 2–4, 4 lateral setae. Dorsocentrals 5–7, 6; acrostichals 9–13, 11; prealars 2–3, 3. Scutellum with 2–4, 4 setae. Humeral pit moderately large, ovoid.

*Legs*. Spur of fore tibia 23–40, 35 μm long; spurs of mid tibia 13–25, 15 μm and 10–18, 12 μm long; spurs of hind tibia 38–50, 41 μm and 10–20, 15 μm long. Hind tibial comb with 9–12, 10 spines, 20–48, 31μm long. Width at apex of fore tibia 35–45, 41 mm, of mid tibia 33–45, 41 mm, of hind tibia 30–45, 38 mm. Lengths (in μm) and proportions of legs as in Table 1.

**Table 1.** Lengths (in μm) and proportions of legs of *Rheocricotopus (Psilocricotopus) brochus* sp. n.

	P_1_	P_2_	P_3_
fe	530–924, 662	530–800, 713	570–820, 629
ti	640–860, 713	530–690, 670	660–870, 724
ta_1_	570–770, 618	330–450, 362	410–550, 446
ta_2_	310–420, 343	150–200, 161	180–260, 205
ta_3_	210–300, 240	100–130, 110	150–210, 169
ta_4_	150–200, 163	50–120, 63	70–100, 83
ta_5_	70–95, 81	55–70, 57	60–80, 69
LR	0.83–0.90, 0.88	0.61–0.65, 0.63	0.60–0.63, 0.61
BV	2.32–2.52, 2.41	3.94–4.62, 4.10	3.30–3.49, 3.42
SV	2.07–2.32, 2.20	3.11–3.48, 3.26	2.95–3.07, 3.02
BR	2.00–4.00, 2.98	1.57–3.00, 2.51	2.25–3.25, 2.67

*Hypopygium* (Figs 5–6). Anal point tapering to apex, 33–41, 37 μm long, 25–50, 37 μm wide in base, with 3–4, 4 lateral setae in each side. Laterosernite IX with 1–3, 2 setae. Phallapodeme 35–68, 55 μm long. Transverse sternapodeme 35–88, 59 μm long. Gonocoxite 138–191, 157 μm long. Superior volsella triangular, 20–40, 31 μm long, with 5–8, 7 setae. Gonostylus 60–75, 66 μm long. Megaseta 9–13, 11 μm long. Crista dorsalis tooth-shaped. HR 2.11–2.60, 2.38. HV 2.59–3.60, 3.31.

#### Type material.

Holotype: ♂ (BDN. C11A32), China, Zhejiang Province, Yueqing City, Lingdi County, Jiulong Village, 28°31'00"N, 120°96'00"E, 18.iv.2011, sweeping, Lin XL. Paratypes (11): 4♂♂, as holotype; 6♂♂, Hubei Province, Hefeng County, 29°91'00"N, 110°03'00"E, 16.vii.1999, light trap, Ji BC; 1♂, Jiangxi Province, Wuyi Mountain National Nature Reserve, 27°48'11"N, 117°39'30"E, 13.vi.2004, light trap, Yan CC.

#### Etymology.

The specific name is an adjective, from Latin *brochus*, meaning tooth, referring to tooth-shaped crista dorsalis.

#### Remarks.

The new species resembles *Rheocricotopus (Psilocricotopus) bifasciatus* Wang & Zheng, 1991 and *Rheocricotopus (Psilocricotopus) insularis* Makarchenko & Makarchenko, 2005 in the following combination of characters: anal point long, pointed distally; wing anal lobe reduced; crista dorsalis tooth-shaped. But the new species can be separated from the latter species in the basis following combination of characters in Table 2.

**Table 2.** Main differences between *Rheocricotopus (Psilocricotopus) brochus* sp. n., *Rheocricotopus (Psilocricotopus) bifasciatus* and *Rheocricotopus (Psilocricotopus) insularis*.

	*Rheocricotopus (Psilocricotopus) brochus* sp. n.	*Rheocricotopus (Psilocricotopus) bifasciatus*	*Rheocricotopus (Psilocricotopus) insularis*
AR	0.63–0.89, 0.72	0.90	0.71–0.74
Length of costal extension	45–65, 51 μm	83 μm	96 μm
Squama	1–2, 2 setae	2 setae	8 setae
Shape of humeral pit	medium, oviod	large, rounded	oviod
Tergite coloration	TI, II, IV yellow, TIII with a brown circular area, others brown	TI, II, IV yellow, others brown	all tergites brown

Female and immature stages unknown.

### Rheocricotopus (Psilocricotopus) chalybeatus

(Edwards, 1929)

http://species-id.net/wiki/Rheocricotopus_chalybeatus

Spaniotoma chalybeatus Edwards, 1929: 331.
Eukiefferiella urbanus Goetghebuer, 1932: 101.
Trichocladius lerutbi Goetghebuer, 1939: 2.
Rheocricotopus chalybeatus Lehmann, 1969: 354; Hirvenoja 1973: 340; Langton 1984: 98.
Rheocricotopus (Psilocricotopus) chalybeatus Sæther, 1985: 82; Wang 2000: 639, Ashe and O’Connor 2012: 561.

#### Specimens examined.

2♂♂, Liaoning Province, Dandong City, Fengcheng City, Cao River, 40°62'50"N, 124°06'96"E, 1.iv.1993, sweeping, Wang JC; 4♂♂, Gansu Province, Longnan City, Gankang County, 33°33'10"N, 105°90'31"E, 2.viii.1982, sweeping, Bu WJ; 1♂, Shandong Province, Zaozhuang City, Shanting District, Beizhuang Town, 34°99'20"N, 102°52'32"E, 28.v.1994, sweeping, Wang XH; 1♂, Shandong Province, Yantai City, Muping District, 37°38'62"N, 121°59'57"E, 28.viii.1988, sweeping, Li HY; 1♂, Shaanxi Province, Baoji City, Feng County, Tsinling Mountains, 34°23'44"N, 106°90'01"E, 27.vii.1994, sweeping, Ji BC; 3♂♂, Zhejiang Province, Wenzhou City, Yueqing City, Furong Town, 33°64'03"N, 121°02'73"E, 2.viii.2010, light trap, Lin XL.

#### Diagnosis.

This species can be separated from its congeners by the following combination of characters: AR 0.89–1.15; R with 2–4 setae; squama with 8–14 setae; Costa not produced or scarcely produced.

#### Remarks.

The additional specimens mainly agree with the description of Lehmann and other workers. But costal extension of specimens from Oriental Region (35–40 μm long) longer than from Palearctic Region (0–15 μm long).

#### Distribution.

China (Liaoning, Gansu, Shandong, Shaanxi and Zhejiang Provinces), Algeria, Balearic Islands, Belarus, Belgium, Corsica, Czech Republic, Denmark, Finland, France, Germany, Great Britain, Hungary, Ireland, Italy, Japan, Lebanon, Luxembourg, Mongolia, Morocco, Netherlands, Poland, Portugal, Romania, Russia, Slovakia, Spain, Switzerland, Syria, Tunisia, Turkey, Ukraine.

### Rheocricotopus (Psilocricotopus) emeiensis

Wang & Zheng, 1989

http://species-id.net/wiki/Rheocricotopus_emeiensis

Rheocricotopus emeiensis Wang & Zheng, 1989: 311.
Rheocricotopus (Psilocricotopus) emeiensis Wang, 2000: 639, Ashe and O’Connor 2012: 563.

#### Specimens examined.

**Type material:** Holotype, ♂, Sichuan Province, Leshan City, Emei Mountain, 29°58'18"N, 103°29'15"E, 17.v.1986, sweeping, Wang XH.

**Additional material:** 1♂, Xinjiang Uygur Autonomous Region, Haba County, 48°17'00"N, 86°42'00"E, 15.vii.2002, sweeping, Tang HQ; 2♂♂, Shaanxi Province, Hanzhong City, Liuba County, Xiaoliuba Village, 33°64'03"N, 106°90'31"E, 4.viii.1994, sweeping, Ji BC; 2♂♂, Shaanxi Province, Xi’an City, Zhouzhi County, Banfangzi, 33°81'84"N, 107°99'64"E, 7.viii.1994, light trap, Ji BC; 2♂♂, Shaanxi Province, Xi’an City, Zhouzhi County, Banfangzi, 33°81'84"N, 107°99'64"E, 10.viii.1994, light trap, Ji BC; 1♂, Yunnan Province, Kunming City, Fumin County, 25°22'61"N, 102°52'32"E, 1.vi.1996, sweeping, Wang XH; 1♂, Yunnan Province, Kunming City, Yiliang County, 24°92'24"N, 103°13'95"E, 2.vi.1996, sweeping, Wang XH; 1♂, Fujian Province, Xiamen City, 24°48'24"N, 118°08'44"E, 15.v.1993, sweeping, Bu WJ; 1♂, Guizhou Province, Guiyang City, 26°60'17"N, 106°70'36"E, 10.vii.1995, light trap, Bu WJ; 1♂, Guizhou Province, Guiyang City, Huaxi District, 26°41'34"N, 106°66'66"E, 23.vii.1995, sweeping, Bu WJ.

#### Diagnosis.

This species can be separated from other members of the group by the following combination of characters: R bare; anal point short, pointed distally.

#### Remarks.

The additional specimens are similar to the description of Wang and Zheng (1989). The species is recorded from Palearctic Region for the first time.

#### Distribution.

China (Fujian, Guizhou, Sichuan, Shaanxi and Yunnan Provinces, Xinjiang Uygur Autonomous Region)

### Rheocricotopus (Psilocricotopus) imperfectus

Makarchenko & Makarchenko, 2005

http://species-id.net/wiki/Rheocricotopus_imperfectus

Rheocricotopus (Psilocricotopus) imperfectus Makarchenko & Makarchenko, 2005: 126; Makarchenko and Makarchenko 2011: 120, Ashe and O’Connor 2012: 564.

#### Specimens examined.

1♂, Hubei Province, Shennongjia Forest Region, 31°74'56"N, 110°67'53"E, 19.vii.1997, sweeping, Du YZ; 1♂, Hubei Province, Lichuan City, 30°29'37"N, 108°93'20"E, 30.vii.1999, sweeping, Ji BC; 12♂♂, Shaanxi, Baoji City, Feng County, Tsinling Mountains, 34°23'44"N, 106°90'01"E, 28–30.vii.1994, sweeping, Bu WJ; 1♂, Shaanxi, Ankang City, Ningshan County, Huoditang Town, 33°43'38"N, 108°44'81"E, 12.viii.1994, sweeping, Bu WJ; 1♂, Shaanxi, Ankang City, Ningshan County, Xunyangba Town, 33°54'82"N, 108°54'77"E, 17.viii.1994, sweeping, Bu WJ; 10♂♂, Ningxia Hui Autonomous Region, Guyuan City, Jingyuan County, Liupan Mountain, 35°78'97"N, 106°28'93"E, 6–7.viii.1987, sweeping, Wang XH.

#### Diagnosis.

This species can be separated from other members of the group by the following combination of characters: AR 0.47; humeral pit large and rounded; acrostichal absent; anal point of hypopygium sharply triangular, with 10 setae along the edges; gonostylus slightly curved, with roundish triangular crista dorsalis.

#### Remarks.

Chinese specimens mainly agree with the description of Makarchenko and Makarchenko (2005), but Chinese specimens with more setae in R (4–8) than the specimens in Russia (R with 3 setae).

#### Distribution.

China (Hubei and Shaanxi Provinces, Ningxia Hui Autonomous Region), Russia (Far East).

### Rheocricotopus (Psilocricotopus) nigrus

Wang & Zheng, 1989

http://species-id.net/wiki/Rheocricotopus_nigrus

Rheocricotopus nigrus Wang & Zheng, 1989: 311.
Rheocricotopus (Psilocricotopus) nigrus Wang, 2000: 639; Makarchenko and Makarchenko 2011: 120, Ashe and O’Connor 2012: 566.

#### Specimens examined.

**Type material:** Holotype, ♂, Hubei Province, Xiangyang City, Gucheng County, 32°29'00"N, 111°64'00"E, 5.v.1986, sweeping, Wang XH; Paratype (1): 1♂, as holotype.

**Additional material:** 2♂♂, Zhejiang Province, Qingyuan County, Baishanzu National Nature Reserve, 27°73'23"N, 119°19'06"E, 18.iv.1995, sweeping, Ji BC; 11♂♂, Xinjiang Uygur Autonomous Region, Mongolian Autonomous Prefecture of Bayingolin, Kuerleying Town, 41°74'10"N, 86°10'82"E, 26.viii.2002, light trap, Tang HQ; 1♂, Xinjiang Uygur Autonomous Region, Mongolian Autonomous Prefecture of Bayingolin, Yuli County, 41°57'00"N, 86°30'00"E, 25.v.2002, sweeping, Tang HQ; 1♂, Xinjiang Uygur Autonomous Region, Haba County, 48°17'00"N, 86°42'00"E, 15.vii.2002, sweeping, Tang HQ; 1♂, Fujian Province, Nanping City, Mangdang Mountain, 26°69'52"N, 118°12'55"E, 22.ix.2002, sweeping, Liu Z; 1♂, Fujian Province, Nanping City, Mangdang Mountain, 26°69'52"N, 118°12'55"E, 23.ix.2002, sweeping, Liu Z.

#### Diagnosis.

The adult male can be separated from other members of the group by the following combination of characters: body totally dark brown; AR 1.30; dorsocentrals 20; humeral pit large, similar to the square.

#### Remarks.

The species distributed in both Oriental and Palearctic Region. The specimens from Palearctic Region have fewer dorsocentrals (13–17) than those from Oriental Region (20).

#### Distribution.

China (Hubei, Zhejiang and Fujian Provinces, Xinjiang Uygur Autonomous Region), Russia (Far East).

### Rheocricotopus (Psilocricotopus) robacki

(Beck & Beck, 1964)

http://species-id.net/wiki/Rheocricotopus_robacki

Tricocladius robacki Beck & Beck, 1964: 204.
Rheocricotopus kenorensis Sæther, 1969: 88.
Rheocricotopus (Psilocricotopus) robacki Sæther, 1985: 79, Ashe and O’Connor 2012: 567.

#### Specimens examined.

1♂, Shaanxi Province, Xi’an City, Zhouzhi County, Banfangzi, 33°81'84"N, 107°99'64"E, 10.viii.1994, light trap, Ji BC; 8♂♂, Jiangxi Province, Yichun City, Yifeng County, 28°39'69"N, 114°67'55"E, sweeping, Yan CC; 1♂, Fujian Province, Longyan City, Shanghang County, 25°05'21"N, 116°41'52"E, 6.v.1993, sweeping, Wang XH; 2♂♂, Xinjiang Uygur Autonomous Region, Boertala Mongol Autonomous Prefecture, Sailimu lake, 44°62'32"N, 81°20'48"E,30.vii.2002, sweeping, Tang HQ; 7♂♂, Guizhou Province, Zunhua City, Daozhen County, Dasha River, 28°86'58"N, 107°60'73"E, 25.v.2004, light trap, Tang HQ; 2♂♂, Guizhou Province, Zunhua City, Daozhen County, Dasha River, 28°86'58"N, 107°60'73"E, 24.viii.2004, sweeping, Yu X; 3♂♂, Yunnan Province, Dali Bai Autonomous Prefecture, Eryuan County, Niujie Town, 26°25'55"N, 99°98'90"E, light trap, Wang BX; 1♂, Tibet, Xigaze, Nielamu County, 27°98'73"N, 85°98'32"E, 15.8.1987, light trap, Deng CY; 1♂, Tibet, Xigaze, Nielamu County, 27°98'73"N, 85°98'32"E, 21.9.1987, light trap, Deng CY.

#### Diagnosis.

The species is characterized by having a relatively high AR (1.14–1.24), very weak and short acrostichals, 8–14 dorsocentrals, reduced number of bristles on squama, anal tergite extending beyond tip of anal point, superior volsella triangular, crista dorsalis triangular, apex pointed.

#### Remarks.

Chinese specimens mainly agree with the description of Sæther (1969, 1985). It is recorded in Palearctic Region for the first time. Chinese specimens have lower body length (2.55–3.13 mm) and lower AR 1.07 than species from Nearctic Region (total length 3.10–3.30 mm, AR 1.18).

#### Distribution.

China (Fujian, Guizhou, Jiangxi, Shaanxi and Yunnan Provinces, Xinjiang Uygur Autonomous Region, Tibet), Canada, U.S.A.

### Rheocricotopus (Psilocricotopus) rotundus sp. n.

http://zoobank.org/5796E5A3-6914-462C-97E2-9CE1709E522E

http://species-id.net/wiki/Rheocricotopus_rotundus

Figs 7–11

#### Diagnosis.

The adult male of the new species can be distinguished from known species of the genus by the following combination of characters: low AR 0.25–0.29; superior volsella rounded.

#### Description.

Male (n = 2).

Total length 1.58–1.98 mm. Wing length 0.86–1.20 mm. Total length/wing length 1.66–1.84. Wing length/length of profemur 1.74–2.61.

*Coloration*. Head and abdomen yellow brown, thorax without distinct pattern.

**Figures 7–11.**
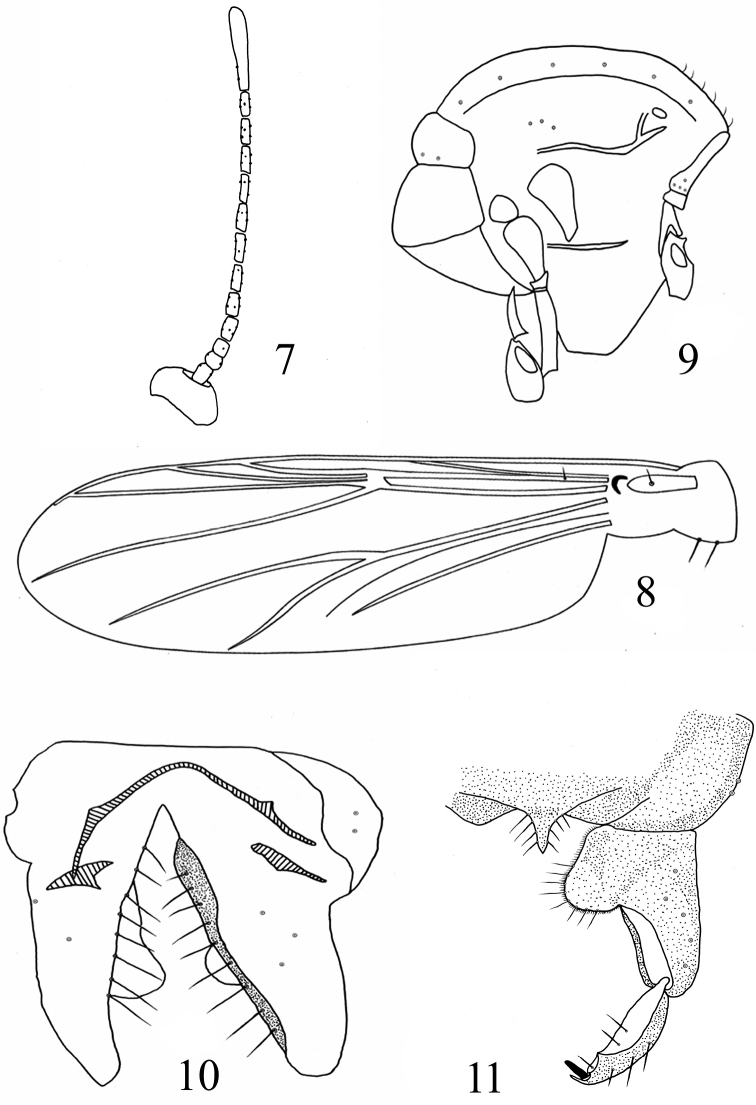
*Rheocricotopus (Psilocricotopus) rotundus* sp. n., male. **7** antenna **8** wing **9** thorax **10** hypopygium (ventral view) **11** hypopygium (dorsal view).

*Head*. Antenna as in Fig. 7. AR 0.25–0.29. Ultimate flagellomere 88–118 μm long. Temporal setae 3–4, including 1–2 inner verticals and 2 outer verticals. Clypeus with 6–12 setae. Tentorium 115–130 μm long, 23–25 μm wide. Stipes 115–118 μm long, 4–5 μm wide. Palpomere lengths (in μm): 48–53, 30–45, 48–60, 68–90, 123–163. L: 5^th^/3^rd^ 2.56–2.71.

*Wing* (Fig. 8). Anal lobe normally developed. VR 1.17–1.19. Costal extension 30–38 μm long. Brachiolum with 1 seta. R with 1–3 setae. Remaining veins bare. Squama with 2 setae.

*Thorax* (Fig. 9). Antepronotum with 4 lateral setae. Dorsocentrals 6–11; acrostichals 6–8, prealars 3. Scutellum with 2–6 setae. Humeral pit moderately large, ovoid.

*Legs*. Spur of fore tibia 23–40 μm long; spurs of mid tibia 13–18 μm long and 12–15 μm long; spurs of hind tibia 27–38 μm and 13–15 μm long. Hind tibial comb with 8–16 spines, 13–27 μm long. Width at apex of fore tibia 25–40 mm, of mid tibia 25–38 mm, of hind tibia 23–40 mm. Lengths and proportions of legs as in Table 3.

**Table 3.** Lengths (in μm) and proportions of legs of *Rheocricotopus (Psilocricotopus) rotundus* sp. n.

	P_1_	P_2_	P_3_
fe	330–490	350–475	310–445
ti	380–500	310–485	350–520
ta_1_	218	135–240	188–300
ta_2_	153	80–135	108–165
ta_3_	120	65–110	90–145
ta_4_	75	35–50	35–65
ta_5_	50	40–50	50–65
LR	0.57	0.44–0.49	0.54–0.58
BV	2.33	3.48–3.61	2.88–3.00
SV	3.26	4.00–4.89	3.22–3.51
BR	2.67	2.00–2.80	3.06–3.83

*Hypopygium* (Figs 10–11). Anal point triangular, 20–28 μm long, with 4 lateral setae each side. Laterosernite IX with 2 setae. Phallapodeme 48–50 μm long. Transverse sternapodeme arcuate with normally oral projection, 33–40 μm long. Gonocoxite 125–135 μm long. Superior volsella rounded, plate-shaped and sclerotized, 28–38 μm long and 20–27 μm wide, covered with 8 short setae and a few microtrichia. Gonostylus 55–70 μm long, with distinct, triangular crista dorsalis. Megaseta 8–10 μm long. HR 1.93–2.27. HV 2.82–2.87.

#### Type material.

Holotype: ♂ (BDN. G5A42), China, Zhejiang Province, Jinhua City, Pan’an County, Dapanshan National Nature Reserve, 28°98'02"N, 120°52'63"E, 18.vii.2012, sweeping, Lin XL. Paratype (1): 1♂, Yunnan Province, Dali Bai Autonomous Prefecture, Eryuan County, Niujie Town, 26°25'55"N, 99°98'90"E, sweeping, Wang BX.

#### Etymology.

The specific name is an adjective, from Latin *rotundus*, meaning rounded, referring to rounded superior volsella.

#### Remarks.

The new species resembles *Rheocricotopus (Psilocricotopus) notabilits* Caspers, 1987 in the following combination of characters: low AR; humeral pit medium, ovoid; the shape of anal point. But the new species can be separated from latter species on the basis of following characters: (1) costal extension of the new species (30–38 μm) much shorter than *Rheocricotopus (Psilocricotopus) notabilits* Caspers (72 μm); (2) anal lobe of the new species developed, which reduced in *Rheocricotopus (Psilocricotopus) notabilits* Caspers; and (3) crista dorsalis of the new species distinct, triangular subapical, which pronounced, rounded in *Rheocricotopus (Psilocricotopus) notabilits* Caspers.

Female and immature stages unknown.

### Rheocricotopus (Psilocricotopus) serratus sp. n.

http://zoobank.org/D6FD7A2F-87EB-4513-A6D4-F122B14548DA

http://species-id.net/wiki/Rheocricotopus_serratus

Figs 12–15

#### Diagnosis.

The adult male of the new species can be distinguished from known species of the species group and the genus by the following combination of characters: crista dorsalis sawtooth-shaped, hyaline, high as megaseta; high HR (2.61–3.42) and HV (3.40–5.00).

#### Description.

Male imago (n = 4)

Total length 2.55–3.00, 2.73 mm. Wing length 1.33–1.95, 1.69 mm. Total length/wing length 1.44–1.94, 1.64. Wing length/length of profemur 2.21–2.35, 2.27.

*Coloration*. Head and abdomen yellow, thorax dark brown.

*Head*. AR 0.71–0.78, 0.74. Ultimate flagellomere 285–355, 323 μm long. Temporal setae 0–3, 1, including 0–1, 1 inner vertical; 0–2, 1 outer vertical and 0–1, 1 postorbital. Clypeus with 9–12, 10 setae. Tentorium 140–153, 148 μm long, 33–40, 38 μm wide. Stipes 65–75, 68 μm long, 3–7, 4 μm wide. Palpomere lengths (in μm): 35–70, 47; 48–58, 53; 103–123, 114; 118–155, 141; 205–238, 219. L: 5^th^/3^rd^ 1.67–2.02, 1.92.

**Figures 12–15.**
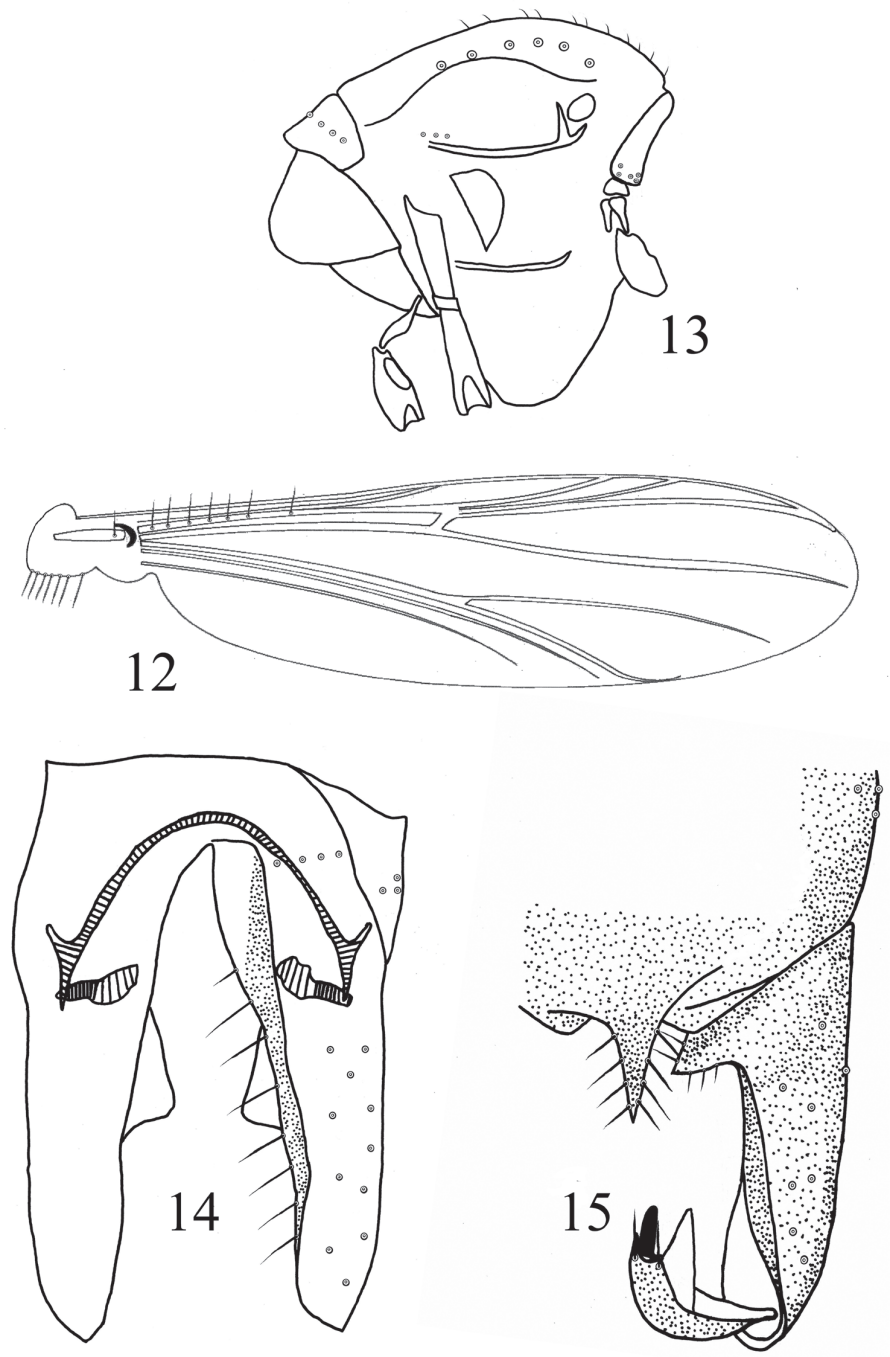
*Rheocricotopus (Psilocricotopus) serratus* sp. n., male. **12** wing **13** thorax **14** hypopygium (ventral view) **15** hypopygium (dorsal view).

*Wing* (Fig. 12). Anal lobe slightly developed. VR 1.13–1.20, 1.17. Costal extension 50–88, 71 μm long. Brachiolum with 1 seta. R with 4–11, 7 setae. Remaining veins bare. Squama with 6–13, 9 setae.

*Thorax* (Fig. 13). Antepronotum with 1–7, 4 lateral setae. Dorsocentrals 6–9, 7; acrostichals 9–15, 12; prealars 3. Scutellum with 4–6, 5 setae. Humeral pit moderately large, egg-shaped.

*Legs*. Spur of fore tibia 40–48, 44 μm long; spurs of mid tibia 15–25, 19 μm and 15–20, 17 μm long; spurs of hind tibia 43–55, 48 μm and 18–23, 19 μm long. Hind tibial comb with 11–13, 12 spines, 30–50, 40 μm long. Width at apex of fore tibia 35–45, 41 mm, of mid tibia 36–45, 41 mm, of hind tibia 38–45, 43 mm. Lengths (in μm) and proportions of legs as in Table 4.

**Table 4.** Lengths (in μm) and proportions of legs of *Rheocricotopus (Psilocricotopus) serratus* sp. n.

	P_1_	P_2_	P_3_
fe	600–830, 743	570–800, 700	600–780, 715
ti	650–810, 760	660–720, 698	660–850, 783
ta_1_	520–780, 640	310–410, 380	380–530, 475
ta_2_	290–390, 350	150–200, 183	200–270, 253
ta_3_	205–270, 250	100–140, 125	160–220, 198
ta_4_	155–200, 181	55–80, 71	90–120, 105
ta_5_	90–100, 95	60–80, 73	70–90, 83
LR	0.78–0.96, 0.84	0.47–0.58, 0.54	0.58–0.63, 0.61
BV	2.39–2.55, 2.44	3.71–4.22, 3.96	2.17–3.04, 2.81
SV	1.95–2.61, 2.37	3.56–3.97, 3.69	2.92–3.32, 3.17
BR	1.75–2.11, 1.91	2.10–2.22, 2.19	2.40–3.55, 2.99

*Hypopygium* (Figs 14–15). Anal point triangular, pointed distally, 33–50, 41 μm long, 20–30, 24 μm wide, with 4–5, 5 lateral setae each side. Laterosernite IX with 2–3, 3 setae. Phallapodeme 23–28, 25 μm long. Transverse sternapodeme 18–33, 26 μm long. Gonocoxite 180–205, 195 μm long. Superior volsella triangular, 35–43, 40 μm long, with 7–8, 8 setae. Gonostylus 60–75, 67 μm long. Megaseta 13–15, 14 μm long. Crista dorsalis sawtooth-shaped, hyaline, high as megaseta. HR 2.61–3.42, 2.91. HV 3.40–5.00, 4.09.

#### Type material.

Holotype: ♂ (BDN. 10058), China, Yunnan Province, Dali Bai Autonomous Prefecture, Eryuan County, Niujie Town, 26°25'55"N, 99°98'90"E, light trap, Zhou CF. Paratypes (3): 1♂, Zhejiang Province, Qingyuan County, Baishanzu National Nature Reserve, 27°73'23"N, 119°19'06"E, 15.vii.1994, Ji BC; 1♂, Tibet, Xigaze, Nielamu County, 27°98'73"N, 85°98'32"E, 21.9.1987, light trap, Deng CY; 1♂, Sichuan Province, Xiangcheng County, 28°93'44"N, 99°79'72"E, 12.vi.1996, light trap, Wang XH.

#### Etymology.

The specific name is an adjective, from Latin *serratus*, meaning sawtooth, referring to the sawtooth-shaped crista dorsalis.

#### Remarks.

The new species resembles *Rheocricotopus (Psilocricotopus) himalayensis* Chaudhuri & Sinharay, 1983 in the triangular anal point, but it can be separated from the latter species on the basis of following characters: (1) costal extension of the new species much longer (50–88 μm), than *Rheocricotopus (Psilocricotopus) himalayensis* Chaudhuri & Sinharay (25 μm); (2) humeral pit in the new species medium, ovoid, which rounded in the latter species; (3) crista dorsalis sawtooth-shaped, hyaline in the new species, which moderately pronounced in the latter species.

Female and immature stages unknown.

### Rheocricotopus (Psilocricotopus) taiwanensis

Wang, Yan & Maa, 2004

http://species-id.net/wiki/Rheocricotopus_taiwanensis

Rheocricotopus (Psilocricotopus) taiwanensis Wang, Yan & Maa, 2004: 239, Ashe and O’Connor 2012: 567.

#### Specimens examined.

**Type material:** Holotype, ♂, Taiwan Province, Taipei City, Guandu, Wetland, 25°11'56"N, 121°47'14"E, 20.x.1988, sweeping, Maa CJ.

#### Diagnosis.

The adult male can be separated from other members of the group by the following combination of characters: all veins of wing bare; low AR (0.71); squama with 3 setae; very pronounced crista dorsalis.

#### Distribution.

China (Taiwan Province).

### Rheocricotopus (Psilocricotopus) valgus

Chaudhuri & Sinharay, 1983

http://species-id.net/wiki/Rheocricotopus_valgus

Rheocricotopus valgus Chaudhuri & Sinharay, 1983: 402.
Rheocricotopus (Psilocricotopus) valgus Ashe & O’Connor, 2012: 568.

#### Specimens examined.

1♂, Guangdong Province, Fengkai County, Heishiding, 23°30'02"N, 111°55'01"E, 20.iv.1988, sweeping, Wang XH; 1♂, Guangxi Province, Longsheng County, 25°89'26"N, 110°21'21"E, 16.v.1990, sweeping, Wang XH; 1♂, Guangxi Province, Jinxiu County, 24°14'00"N, 110°19'00"E, 1.vi.1990, light trap, Wang XH; 2♂♂, Hubei Province, Hefeng County, 29°91'00"N, 110°03'00"E, 16.vii.1999, light trap, Ji BC; 1♂, Hubei Province, Xianfeng Mountain, 29°70'00"N, 119°14'00"E, 25.vii.1999, sweeping, Ji BC; 1♂, Zhejiang Province, Lishui City, Qingyuan County, Baishanzu, 27°73'23"N, 119°19'06"E, 13.vii.1995, light trap, Ji BC; 4♂♂, Zhejiang Province, Lishui City, Qingyuan County, Baishanzu National Nature Reserve, 27°73'23"N, 119°19'06"E, 24.vii.2012, light trap, Lin XL; 1♂, Zhejiang Province, Wenzhou City, Taishun County, Wuyanling National Nature Reserve, 27°71'15"N, 119°64'64"E, 3.viii.2005, light trap, Ji BC; 1♂, Zhejiang Province, Lishui City, Jingning County, 27°97'67"N, 119°63'12"E, 27.vii.2012, light trap, Lin XL.

#### Diagnosis.

This species can be separated from other members of the group by the following combination of characters: R without seta; tergites I, II and anterior part of tergite V pale brown, tergites IV and VIII brown, anal point with 4–5 setae on each side and 1 seta at the base; gonocoxite with a prominent triangular basal lobe bearing 3–4 setae.

#### Remarks.

The Chinese specimens generally agree with the original description by Chaudhuri and Sinharay (1983), though some measured differences between the Chinese specimens and those of Chaudhuri and Sinharay (1983) are shown in Table 5.

**Table 5.** Differences in the Chinese and Indian specimens of *Rheocricotopus (Psilocricotopus) valgus*

	Chinese specimens	Indian specimens
Squama	5–8, 7 setae	9 setae (average)
HR	1.98–2.15, 2.08	2.20 (average)
HV	2.88–3.20, 3.03	3.40 (average)

#### Distribution.

China (Guangdong, Guangxi, Hubei and Zhejiang Provinces), India.

### Key to adult males of *Rheocricotopus chalybeatus* species group in China

**Table:** 

1	Anal lobe reduced	2
–	Anal lobe moderately or very developed	5
2	Costa not beyond R_4+5_	*Rheocricotopus (Psilocricotopus) robacki* (Beck & Beck)
–	Costa beyond R_4+5_	3
3	Squama with 9 setae	*Rheocricotopus (Psilocricotopus) bifasciatus* Wang & Zheng
–	Squama bare, or with 1 seta	4
4	Tergites I, II, IV yellow, tergite III with a brown circular area, others brown	*Rheocricotopus (Psilocricotopus) brochus* sp. n.
–	All tergites I-IX dark brown	*Rheocricotopus (Psilocricotopus) imperfectus* Makarchenko & Makarchenko
5	AR 0.25–0.29; superior volsella rounded	*Rheocricotopus (Psilocricotopus) rotundus* sp. n.
-	AR 0.45–1.30; superior volsella triangular	6
6	Humeral pit large, similar to the square	7
–	Humeral pit rounded or ellipsoid	8
7	Dorsocentrals 20; R_1_ with 1 seta	*Rheocricotopus (Psilocricotopus) nigrus* Wang & Zheng
–	Dorsocentrals 10–14; R_1_ bare	*Rheocricotopus (Psilocricotopus) chalybeatus* (Edwards)
8	Crista dorsalis sawtooth-shaped, transparent	*Rheocricotopus (Psilocricotopus) serratus* sp. n.
–	Crista dorsalis triangular or rectangular	9
9	Costal extension 120 μm long; AR 1.20	*Rheocricotopus (Psilocricotopus) valgus* Chaudhuri & Sinharay
–	Costal extension 30–45 μm long; AR 0.43–1.00	10
10	Acrostichals absent	11
–	Acrostichals 7–9	*Rheocricotopus (Psilocricotopus) brachypus* Wang & Zheng
11	Supraalars present; crista dorsalis rectangular	*Rheocricotopus (Psilocricotopus) emeiensis* Wang & Zheng
–	Supraalars absent; crista dorsalis triangular	*Rheocricotopus (Psilocricotopus) taiwanensis* Wang, Yan & Ma

## Supplementary Material

XML Treatment for Rheocricotopus (Psilocricotopus) bifasciatus

XML Treatment for Rheocricotopus (Psilocricotopus) brachypus

XML Treatment for Rheocricotopus (Psilocricotopus) brochus

XML Treatment for Rheocricotopus (Psilocricotopus) chalybeatus

XML Treatment for Rheocricotopus (Psilocricotopus) emeiensis

XML Treatment for Rheocricotopus (Psilocricotopus) imperfectus

XML Treatment for Rheocricotopus (Psilocricotopus) nigrus

XML Treatment for Rheocricotopus (Psilocricotopus) robacki

XML Treatment for Rheocricotopus (Psilocricotopus) rotundus

XML Treatment for Rheocricotopus (Psilocricotopus) serratus

XML Treatment for Rheocricotopus (Psilocricotopus) taiwanensis

XML Treatment for Rheocricotopus (Psilocricotopus) valgus
